# Extracts of Pomelo Peels Prevent High-Fat Diet-Induced Metabolic Disorders in C57BL/6 Mice through Activating the PPARα and GLUT4 Pathway

**DOI:** 10.1371/journal.pone.0077915

**Published:** 2013-10-16

**Authors:** Xiaobo Ding, Lu Guo, Yu Zhang, Shengjie Fan, Ming Gu, Yan Lu, Dong Jiang, Yiming Li, Cheng Huang, Zhiqin Zhou

**Affiliations:** 1 School of Pharmacy, Shanghai University of Traditional Chinese Medicine, Shanghai, China; 2 College of Horticulture and Landscape Architecture, Southwest University, Chongqing, China; 3 Key Laboratory of Horticulture Science for Southern Mountainous Regions, Ministry of Education, Chongqing, China; 4 Citrus Research Institute, Chinese Academy Agricultural Science, Chongqing, China; University of North Dakota, United States of America

## Abstract

**Objective:**

Metabolic syndrome is a serious health problem in both developed and developing countries. The present study investigated the anti-metabolic disorder effects of different pomelo varieties on obese C57BL/6 mice induced by high-fat (HF) diet.

**Design:**

The peels of four pomelo varieties were extracted with ethanol and the total phenols and flavonoids content of these extracts were measured. For the animal experiment, the female C57BL/6 mice were fed with a Chow diet or a HF diet alone or supplemented with 1% (w/w) different pomelo peel extracts for 8 weeks. Body weight and food intake were measured every other day. At the end of the treatment, the fasting blood glucose, glucose tolerance and insulin (INS) tolerance test, serum lipid profile and insulin levels, and liver lipid contents were analyzed. The gene expression analysis was performed with a quantitative real-time PCR assay.

**Result:**

The present study showed that the *Citrus grandis* liangpinyou (LP) and beibeiyou (BB) extracts were more potent in anti-metabolic disorder effects than the duanshiyou (DS) and wubuyou (WB) extracts. Both LP and BB extracts blocked the body weight gain, lowered fasting blood glucose, serum TC, liver lipid levels, and improved glucose tolerance and insulin resistance, and lowered serum insulin levels in HF diet-fed mice. Compared with the HF group, LP and BB peel extracts increased the mRNA expression of PPARα and its target genes, such as FAS, PGC-1α and PGC-1β, and GLUT4 in the liver and white adipocyte tissue (WAT).

**Conclusion:**

We found that that pomelo peel extracts could prevent high-fat diet-induced metabolic disorders in C57BL/6 mice through the activation of the PPARα and GLUT4 signaling. Our results indicate that pomelo peels could be used as a dietary therapy and the potential source of drug for metabolic disorders.

## Introduction

Metabolic syndrome is a growing health problem worldwide and is characterized by multiple metabolic disorders, such as obesity, hyperglycemia, hyperlipidemia, fatty liver and insulin resistance [1]. Peroxisome proliferator-activated receptors (PPARs), the ligand-activated transcription factors, are the regulators of the glucose metabolism, adipocyte differentiation and lipogenesis [2]. The PPAR family consists of PPARα, PPARγ, and PPARδ/β. PPARα, the first PPAR isoform to be identified, is involved in regulating lipid metabolism in the liver and skeletal muscle [3]. Ligands of PPARα have been used for fighting metabolic syndrome. For example, fibrates, the PPARα agonist, have been in clinical use for the treatment of dyslipidemia [4]. Activation of PPARα lowers plasma triglycerides (TG) and increases plasma high density lipoprotein cholesterol (HDL-c) levels [5]. PPARα is also an important regulator of carbohydrate metabolism. It has been previously shown that PPARα agonist can improve insulin resistance, and decrease the fasting blood glucose level in obese mice [6,7]. 

Current studies have shown that the natural compounds have effects on PPARα and could be useful for human health [8]. Various citrus peels have been used in Chinese Medicine for centuries to cure indigestion, cough, nausea, cardiovascular diseases, liver diseases, constipation, skin inflammation, cancer and muscle pain [9,10]. Citrus peels are rich in bioactive compounds such as flavonoids, coumarins, limonoids, alkaloids and polyphenols which exhibit a wide range of biological activities, including anti-cardiac, anti-tumor, anti-oxidative, anti-inflammatory, anti-hypertension, anti-hyperglycemia, anti-hyperlipidemia and anti-obesity [11-17]. 

Recently, there has been an increasing interest in the research of citrus peels on the prevention and treatment of metabolic disorders. An *in vitro* study indicated that the *Citrus depressa* Hayata (shiikuwasa) extract had an anti-obesity effect by regulating the expression of lipogenesis-related genes in white adipose tissue [18]. *Citrus tangerina* peel extract has been reported to decrease plasma and hepatic cholesterol levels in rats [19]. Extract from the peel of *Citrus unshiu* has the potential to ameliorate both hyperglycemia and hepatic steatosis in *db/db* mice [20]. Immature *Citrus sunki* peel extract has been shown to block body weight gain and reduce serum levels of TG and total cholesterol (TC) in high-fat diet-induced obese mice [11]. According to our previous study, *Citrus ichangensis* peel extract administration could alleviate obesity and its related metabolic disorders in high-fat diet-fed mice [21]. These potentially health-promoting effects have been ascribed to the citrus flavonoids. Flavonoids are a large group of polyphenolic compounds that are ubiquitous in dietary fruits and vegetables. There have been convincing evidences that high consumption of flavonoids is beneficial in preventing and fighting metabolic syndrome [22]. 

Pomelo (*Citrus grandis* (L.) Osbeck), also known as Chinese grapefruit, is one of the most common fruits in China with many different cultivars. Pomelo belongs to the genus *Citrus* of the family Rutaceae and is the largest citrus fruit. Previous studies showed that pomelo peels contain an abundance of bioactive compounds, such as flavonoid and pectin [23,24]. A study by Hong et al. suggested that Dangyuja (*Citrus grandis* Osbeck) peel could improve lipid profiles and alleviate hypertension in high-fat diet-induced obese rats [25]. It has been reported that pomelo peel extract in obese mice reduced body weight and blood TG, TC, high density lipoprotein cholesterol (LDL-c) by activating major enzymes related to lipid metabolism, such as lipase and carnitine palmitoyl-transferase [26]. The present study aimed to compare the effects of different pomelo peel extracts on high-fat diet-induced obesity, hyperglycemia and dyslipidemia of C57BL/6 mice, and to find the constituent-activity relationship. 

## Material and Methods

### Plant materials and sample preparation

Four varieties of pomelo fruits, *Citrus grandis* ‘Liangpinyou’ (LP), *C. grandis* ‘duanshiyou’ (DS), *C. grandis* ‘wubuyou’ (WB), and *C. grandis* ‘beibeiyou’ (BB) were obtained from the Citrus Research Institute, Chinese Academy of Agricultural Sciences, Chongqing, China. The samples were botanically authenticated by Dr. Jie Yu from Southwest University. 

Pomelo peels were separated from the fruits by hand, dried at 50°C and powdered by mechanical grinder. To obtain extracts, dried peel powder (100 g) was extracted with 90% ethanol (2 L) twice at 80°C for 2 h, followed by filtration with Whatman NO.1 filter paper. After that, the filtered solution was evaporated at 40°C with a rotary evaporator under reduced pressure, freeze-dried to a powder, and stored at -20°C until use.

### Determination of total phenolic content

The total phenolic content of extracts was measured using the Folin-Ciocalteu assay [27]. 0.25 ml of the diluted extract was mixed with 3.5 ml of distilled water and 0.25 ml of Folin-Ciocalteu reagent. After 3 min, 1 ml of 20% sodium carbonate was added. The tubes were kept for 40 min at 40°C before the absorbance was measured at 685 nm with a spectrophotometer. The total phenolic contents were expressed as a gallic acid equivalent in milligram per gram of extracts. 

### Determination of total flavonoid content

The colorimetric assay adapted from Ramful et al. was used for the determination of total flavonoids present in the extracts [27]. 2.5 ml of diluted extract was mixed with 150 μl of 5% aqueous NaNO_2_. After 5 min, 150 μl of 10% aqueous AlCl_3_ was added. After 1 min, 1 ml of 1 M NaOH was added. The absorbance of the whole mixture was read at 510 nm with a spectrophotometer. Results are expressed in milligram of rutin per gram of extracts.

### HPLC analysis

The extracts were diluted with methanol and then filtered with a 0.45-μm syringe filter before analysis by high performance liquid chromatography (HPLC). Quantitative analysis of flavonoid was performed on an Agilent 1200 liquid chromatograph system. The flavonoid compounds were separated on a Discovery C18 HPLC Column (250×4.6 mm, 5 μm) at 30°C. The mobile phase consisted of water containing 0.5% acetic acid (A) and acetonitrile (B) at a flow rate of 1.0 ml/min. The gradient profile was as follows: 0-15 min, 15-25% B; 15-17 min, 25% B; 17-20 min, 25-50% B; 20-35 min, 50-60% B; 35-40 min, 60-85% B; 40-45 min, 85B%; and 45-50 min, back to 15% B. The injection volume was 10 μl, and the diode array detector was set at 280 nm.

### Animals and diets

The animal protocols used in this study were approved by the Shanghai University of Traditional Chinese Medicine for animal studies (Approved Number: 12022). Female C57BL/6 mice were purchased from the SLAC Laboratory (Shanghai, China) at 6 weeks of age. Mice were housed at 23±2°C on a 12 h light/dark cycle. After a one-week adaptation period, animals were randomly divided into six groups (n=7) with different diets. All of the mice were given free access to diet and water for 8 weeks. Body weight and food intake were measured every other day.

### Glucose tolerance and insulin tolerance test

For glucose and insulin (INS) tolerance test, all the mice were fasted for 12 h after 8 weeks treatment of pomelo peel extracts or not. Animals were intraperitoneally injected with either 1 g/kg glucose or 0.75 U/kg INS. The blood samples were collected from the tail vein for measurement of a basal blood glucose levels (0 min) before the injection of glucose or INS. Additional blood glucose levels were measured at 15, 30, 60 and 90 min.

### Serum chemistry analysis

At the end of the experimental period, the mice were anesthetized with urethane following a 12-h fast. Blood samples were drawn from the heart into a vacuum tube, and Serum samples were separated from the blood. Serum TG, TC, LDL-c, and HDL-c were all assayed using a Hitachi 7020 Automatic Analyzer (Hitachi, Ltd., Tokyo, Japan) with 100 μl of heart blood serum. Meanwhile, the plasma INS concentrations were measured using a Mouse Insulin ELISA Kit (LINCO Research, Inc., St. Chalfont, Buckinghamshire, UK) according to the manufacturer’s instructions.

### Liver lipid content analysis

The hepatic lipids were extracted from the liver tissues using the method of Folch, Lees, and Sloan-Stanley [28]. The dried lipid residues were then re-suspended in 100 μl of isopropyl alcohol for TG and TC assays. The hepatic TG and TC levels were estimated with the same method as used in the plasma analysis.

### Quantitative real-time PCR

Total RNA was extracted from mouse tissues using the method of Zhang et al. [29]. The total RNA was reverse transcribed into first-strand cDNA using a cDNA synthesis kit (Promega Corp., Madison, WI) according to the manufacturer’s instructions. The gene expression levels were analyzed by quantitative real-time PCR using the ABI 7500 Real-time PCR system (Applied Biosystems, Foster City, Calif., USA). After an initial incubation for 2 min at 50°C, the cDNA was denatured at 95°C for 10 min, followed by PCR 40 cycles (95°C, 15 sec; 60°C, 60 sec). All results were obtained in at least three independent experiments. The gene mRNA levels were normalized using β-actin as an internal control. The primers involved in the experiments are shown in Table 1.

**Table 1:** Sequences of the primers used in real-time PCR.

Gene	Forward primer	Reverse primer
β-Actin	TGTCCACCTTCCAGCAGATGT	AGCTCAGTAACAGTCCGCCTAGA
PPARα	AGGCTGTAAGGGCTTCTTTCG	GGCATTTGTTCCGGTTCTTC
FAS	CTGAGATCCCAGCACTTCTTGA	GCCTCCGAAGCCAAATGAG
GLUT4	GGCTTTGTGGCCTTCTTTGAG	GACCCATAGCATCCGCAACAT
PGC-1α	TGTTCCCGATCACCATATTCC	GGTGTCTGTAGTGGCTTGATTC
PGC-1β	GGGTGCGCCTCCAAGTG	TCTACAGACAGAAGATGTTATGTGAACAC
ACC	GAATCTCCTGGTGACAATGCTTATT	GGTCTTGCTGAGTTGGGTTAGCT
ACO	CAGCACTGGTCTCCGTCATG	CTCCGGACTACCATCCAAGATG
Cyp4a10	GAGTGTCTCTGCTCTAAGCCCA	AGGCTGGGGTTAGCATCCTCCT
Cyp4a14	TGAATTGCTGCCAGATCCCACCAGGATC	GTTCAGTGGCTGGTCAGA
CD36	GCTTGCAACTGTCAGCACAT	GCCTTGCTGTAGCCAAGAAC

### Statistical analysis

All values are expressed as the mean ± SD unless otherwise indicated. Data analysis was performed by SPSS 12.0 software for Windows statistical program. Statistical analysis was programmed by one-way analysis of variance (ANOVA). Differences were defined as significant when P < 0.05.

## Results

### The total phenolic, total flavonoids and flavonoid contents in pomelo peel extracts

The pomelo peels were extracted and their constituents were tested. The total phenolic and total flavonoid content of the extracts are shown in Table 2. The concentrations of total phenolic ranged from 42.79 to 54.56 mg gallic acid equivalent/g in the following increasing order: WB < DS < BB < LP. The highest level of total flavonoids was obtained in LP extracts (26.70 mg of rutin/g). The second largest concentration of total flavonoids was measured in BB (20.88 mg of rutin/g), followed by DS (20.28 mg of rutin/g). The lowest amount of total flavonoids was found in WB (13.43 mg of rutin/g). The flavonoid profile of the four pomelo peel extracts was analyzed by HPLC, and the flavonoid contents in the extracts are shown in Table 2. Naringin and didymin were detected in all of the pomelo extracts, and naringin was present at the highest concentration in all of them. The highest amount of naringin was measured in LP extract, whereas the highest level of didymin was obtained in BB extract. These extracts were found to have no polymethoxyflavones, such as nobiletin and tangeretin. 

**Table 2:** Total phenolic and flavonoids content of different pomelo variety peel extracts.

Variety	Total phenolic (mg/g)	Flavonoids (mg/g)					
		Total flavonoids	Naringin	Hesperidin	Didymin	Nobiletin	Tangeretin
*C. grandis* ‘Liangpinyou’ (LP)	53.64±1.32	26.70±0.29	17.00±0.34	0.98±0.23	3.90±0.25	ND	ND
*C. grandis* ‘Duanshiyou’ (DS)	46.02±0.77	20.28±0.13	10.29±0.12	ND	2.63±0.17	ND	ND
*C. grandis* ‘Wubuyou’ (WB)	42.79±0.84	13.43±0.28	12.52±0.22	ND	1.56±0.12	ND	ND
*C. grandis* ‘beibeiyou’ (BB)	46.06±0.83	20.88±0.27	12.65±0.31	ND	4.55±0.24	ND	ND

Values are expressed as means ± SEM of three independent experiments. ND = not detected.

### Pomelo peel extracts ameliorate diet-induced obesity and improve glucose tolerance

**Figure 1:**
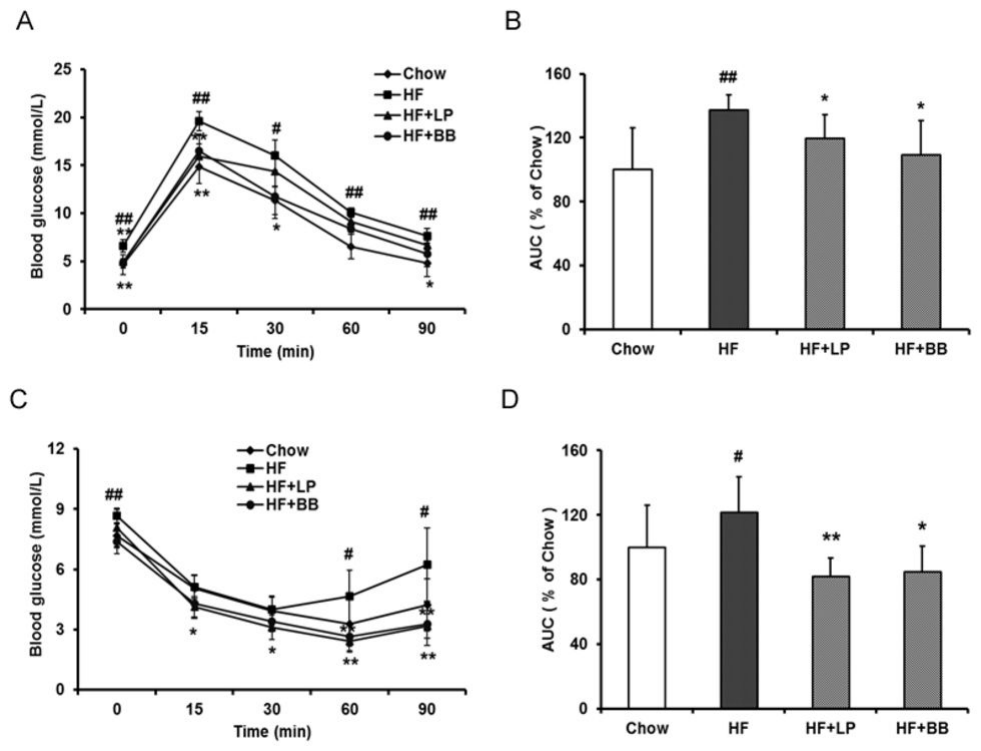
Effects of different pomelo variety peel extracts on glucose intolerance and insulin intolerance in high-diet fed mice. (A) Intraperitoneal glucose tolerance test (IPGTT). The mice were fasted for 12 hours, and the baseline blood glucose was measured at 0 min, and then 1 g glucose/kg body weight was injected intraperitoneally. Glucose levels were tested at regular intervals of 15, 30, 60, and 90 min. (B) Quantification of the area under the curve (AUC) from the GTT in (A); (C) Intraperitoneal insulin tolerance test (IPITT). The non-fasted glucose level (0 min) was measured at 0 min, then the blood glucose levels at 15, 30, 60, and 90 min were tested following intraperitoneal insulin injection (1 U/kg body weight). (D) Quantification of the area under the curve (AUC) from the ITT in (C). Values are expressed as means ± SD. (n=7; #p<0.05, ##p<0.01, vs. the Chow group; *p<0.05, **p<0.01, vs. the HF group).

In order to test the effects of pomelo peel extracts on metabolic disorders, we fed the mice with either chow diet or a high-fat diet for 8 weeks. The obtained results show that the mean body weight of HF diet-fed mice was increased compared to chow diet-fed mice (P<0.01, Table 3). These results showed that the HF diet-fed mice suffer from a carbohydrate metabolism disorder. Table 3 displayed that the final body weight of LP and BB treated mice were lowered markedly compared to that in the HF-fed mice (P<0.05), while the final body weight of DS and WB treated mice remained no significant change. During the treatment, the different groups of mice showed roughly equivalent food intake. The fasting blood glucose concentrations of HF diet-fed mice were increased compared to chow diet-fed mice (p<0.05, Table 3). LP, DS and BB treatment decreased the fasting blood glucose levels significantly. Next, we assayed the glucose tolerance of the mice. As shown in Figure 1A, the blood glucose levels were significantly increased in HF diet-fed mice following the injection of glucose in comparison with chow diet-fed mice. The obtained results showed that BB extract treatment significantly lowered blood glucose levels at 15, 30, and 90 min in comparison with the HF group. At the 15 and 30 min intervals, blood glucose levels were significantly lower in the HF+LP group than in the HF group. The total area under the curve (AUC) of blood glucose levels between 0 to 90 min were 9.07±1.39, 12.47±0.86, 10.88±1.31, and 9.94±1.90 mmol/l/min for the Chow group, HF group, HF+LP group and HF+BB group, respectively, (Figure 1B). Although DS lowered the fasting blood glucose, it did not improve the glucose tolerance at at any point (data not shown). These results demonstrate that both LP and BB peel extracts could improve glucose tolerance in HF diet-fed mice.

**Table 3:** Effects of different pomelo variety peel extracts supplementation on body weight gain, food intake and fasting blood glucose in the high-fat diet-induced obese group after 8 weeks.

Group	Chow	HF	HF+LP	HF+DS	HF+WB	HF+BB
Initial body weight (g)	18.57±0.53	18.57±0.53	18.29±0.76	18.57±0.53	18.57±0.98	18.43±0.53
Final body weight (g)	23.71±0.47	28.71±0.61 ##	26.43±0.81 *	27.00±0.90	30.00±0.95	26.00±0.90 *
Food intake (g/mouse/d)	2.34±0.11	2.18±0.21	2.05±0.17	2.01±0.23	2.15±0.22	2.04±0.17
Fasting blood glucose (mmol/L)	4.63±0.43	6.57±0.66 ##	4.96±0.71 **	5.24±0.82 *	5.53±1.07	4.89±0.36 **

Values are expressed as means ± SEM. (n=7; #p<0.05, ##p<0.01, vs. the Chow group; *p<0.05, **p<0.01, vs. the HF group).

### Pomelo peel extracts improve insulin resistance

**Figure 2:**
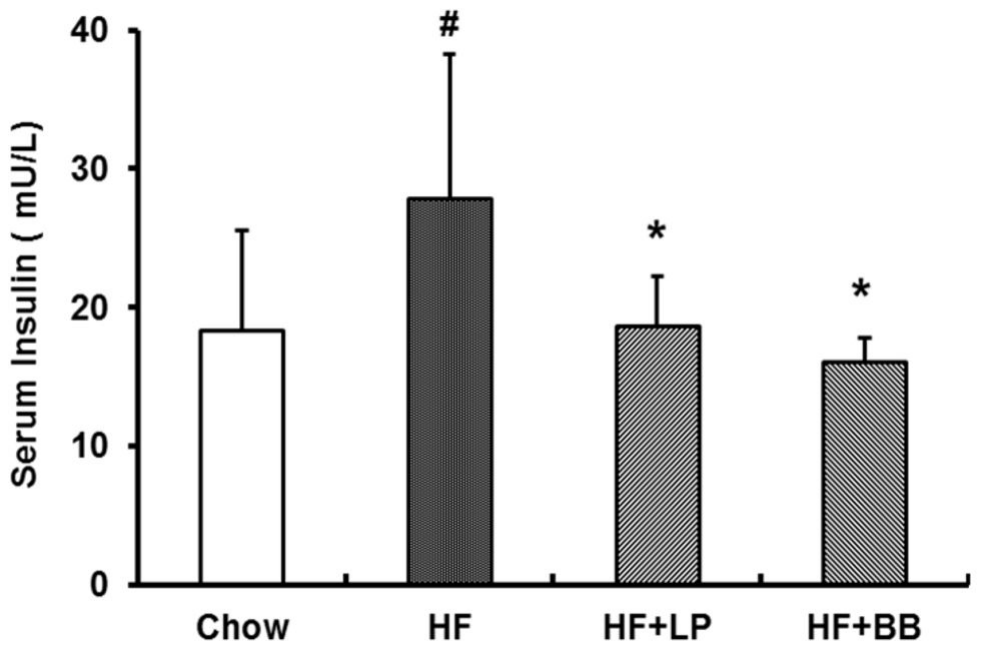
Effects of different pummelo variety peel extracts on serum insulin levels in high-fat diet-fed. Values are expressed as means ± SD. (n=7; #p<0.05 vs. the Chow group; *p<0.05 vs. the HF group).

Since LP and BB extracts significantly decreased fasting blood glucose levels and improved glucose tolerance, we then investigated whether these extract treatments would affect insulin tolerance *in vivo*. The HF diet-fed mice exhibited impaired insulin resistance compared with mice on Chow diet. The blood glucose levels in HF+BB and HF+LP treated mice were markedly lower than that of HF mice (Figure 1C). However, we did not observe that significant change in other extract treatments (data not shown). We then assayed the serum insulin levels of the mice. In the HF diet fed mice, the serum insulin was elevated significantly. LP and BB peel extract administration significantly reduced serum insulin levels in the mice (Figure 2). The total AUC of blood glucose levels between 0 and 90 min were 5.15±0.93, 4.24±1.10, 3.48±0.48 and 3.60±0.66 mmol/l/min for the Chow group, HF group, HF+LP group and HF+BB group, respectively (Figure 1D). These data suggest that the insulin resistance induced by the HF diet was significantly improved by treatment with LP peel extract or BB peel extract.

### Pomelo peel extracts improve the lipid parameters in serum and liver tissue

**Figure 3:**
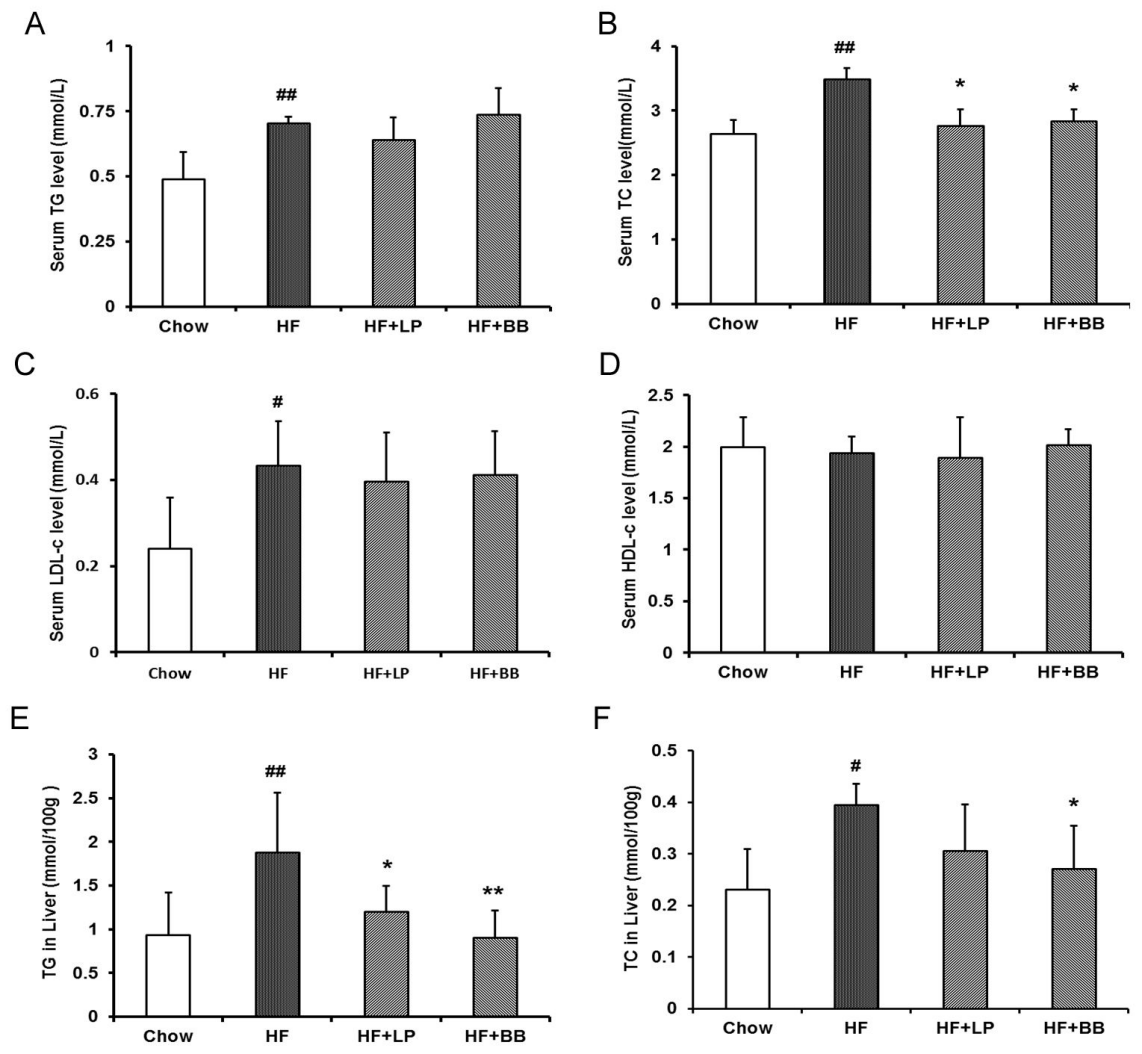
Effects of different pummelo variety peel extracts on serum and liver lipid profiles in high-fat diet-fed mice. (A-D) Serum triglyceride (TG) levels, total cholesterol (TC) levels, low density lipoprotein cholesterol (LDL-c) levels, and high density lipoprotein cholesterol (HDL-c). (E and F) Liver TG and TC contents. Values are expressed as means ± SD. (n=7; #p<0.05, ##p<0.01, vs. the Chow group; *p<0.05, **p<0.01, vs. the HF group).

The lipid levels in serum and liver tissue were measured and are shown in Figure 3. The fasting serum TG, TC, and LDL-c concentrations of the HF diet-fed group were increased 1.44, 1.32, and 1.81 times, respectively, compared to Chow diet-fed mice. There was no significant difference in HDL-c concentrations in the HF group compared with the Chow group. The HF+LP group and HF+BB group showed significantly reduced levels of TC in comparison to the HF group. We did not observe differences in the TG, LDL-c and HDL-c levels in HF+LP group or HF+BB group compared to HF group. These results suggested that pomelo peel extracts may prevent high-fat diet-induced hyperlipidemia, at least partly, by decreasing TC level in serum. Next, we assayed the hepatic TG and TC levels; the results showed that TG and TC were significantly increased in the HF diet-fed mice when compared to the Chow diet-fed mice. Both LP peel extract and BB peel extract treatment markedly decreased the TG levels in the livers of the HF diet-induced mice. Moreover, the TC content was also significantly reduced in the livers of HF+BB group compared with the HF group, whereas the TC content was not significantly changed by LP peel extract treatment. 

### Pomelo peel extracts regulates the expression of metabolic genes

**Figure 4:**
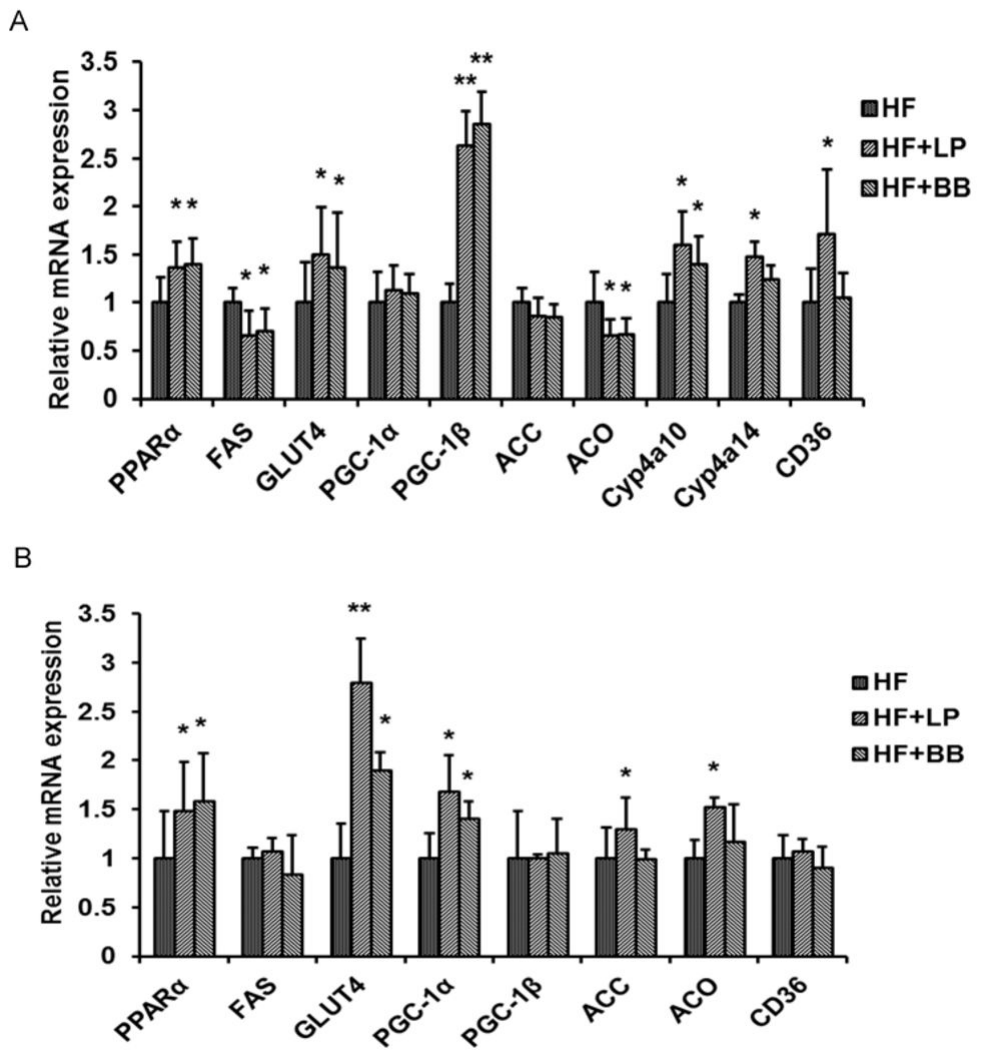
Effects of pomelo peel extracts on the gene expression in liver and WAT. (A) The relative expression levels of genes in the liver tissue. (B) The relative expression levels of genes in the WAT. For real-time PCR, the results were repeated in at least 3 independent experiments. β-actin was used as an internal control for normalizing the mRNA level. Values are expressed as means ± SD. (n=7; *p<0.05, **p<0.01, vs. the HF group).

The beneficial effects of LP and BB peel extracts on glucose and lipid metabolism were reflected by their effects on related gene expression in the liver and white adipocyte tissue (WAT) of mice. In the liver tissue, the mRNA levels of PPARα and its target genes peroxisome proliferator-activated receptor-γ coactivator-1β (PGC-1β) and cytochrome P450, family 4, subfamily a, polypeptide 10 (Cyp4a10) were increased in HF+LP and HF+BB groups relative to the HF control group, whereas the gene expression of Fatty acid synthase (FAS) and Acyl-CoA oxidase (ACO) were markedly inhibited. However, the expression of peroxisome proliferator-activated receptor-γ coactivator-1α (PGC-1α), and Acetyl Coenzyme A carboxylase (ACC) were not changed by treatment with these extracts. In the WAT, the expression levels of PPARα and its target genes ACC, ACO, and PGC-1α in the HF+LP group were increased in comparison with those of the HF group. The expression levels of PPARα, and PGC-1α were also increased in the HF+BB group compared with the HF group. There was no difference in the mRNA expression levels of FAS, Cluster of differentiation 36 (CD36), and PGC-1β in the WAT of extract-treated and untreated mice. Glucose transporter 4 (GLUT4) plays an important role in regulating whole body glucose homeostasis. In the present study, the expression level of GLUT4 was increased in HF+LP and HF+BB groups in comparison to the HF group (Figure 4). These results provide a possible explanation for the anti-metabolic disorder effects of pomelo peel extracts.

## Discussion

In this study, we provided evidence that the anti-metabolic disorder effects among the four pomelo peels were significantly different. Our results suggested that both LP and BB peel extracts have preventive effects against the development of metabolic disorders, including obesity, fatty liver, dyslipidemia and hyperglycemia in mice. Furthermore, we found that the expression of the nuclear receptor transcription factor PPARα was increased in the liver and WAT of mice treated with LP or BB peel extracts. These results suggest that the preventive effects of pomelo peel extracts against metabolic disorders are likely through the enhancement of PPARα signaling.

Pomelo is a popular citrus fruit in China with many different cultivars. Previous studies suggested that pomelo peels are rich source of bioactive compounds such as flavonoid, pectin, phenolic, kaemferol and its derivatives [23,24,30]. Several studies have reported that pomelo peel or pomelo peel extracts have a beneficial effect on metabolic disorders by blocking body weight gain, improving glucose metabolism, and attenuating dyslipidemia [25,26,31]. A number of potentially beneficial effects have been attributed to citrus flavonoids, which are the most important class of polyphenolic compounds [32-35]. For example, hesperedin and naringin both exhibited anti-diabetic properties in high fat-fed/streptozotocin-induced type 2 diabetic rats [36]. There are more than 60 flavonoids that have been isolated and identified from grapefruit, pomelo and other *Citrus* species. A clinical trial has suggested that the citrus flavonoid naringin (400 mg/day) exhibited lipid lowering properties in hypercholesterolemic subjects [33]. The combination of citrus PMF and tocotrienols has been reported to reduce plasma TG, TC and LDL-c in hypercholesterolemic patients [37]. However, in another clinical trial, hesperidin (800 mg/day) and naringin (400 mg/day) did not affect serum cholesterol in moderately hypercholesterolemic individuals [38]. Our data show that the LP and BB extracts had more potent anti-metabolic disorder effects than DS and WB. Because total phenols and flavonoids in DS and BB are similar, their beneficial effects may be dependent on the composition and levels of these bioactive compounds in pomelo peel extracts. In the current study, body weight gain, fasting blood glucose concentration, serum TC and INS levels, and liver lipid levels were significantly increased in the HF group relative to those in the Chow group, and were improved by LP or BB peel extract supplementation. In addition, both LP and BB peel extracts also improved glucose tolerance and insulin resistance or serum insulin contents in the HF diet-induced mice. These anti-metabolic disorder effects observed in the present study support the previous reports that supplementation with pomelo peel extracts exhibited anti-obesity and anti-diabetic effects in experimental obese and type 2 diabetic mice [26]. We noticed that the contents of didymin in LP and BB are higher than that in DS and WB. Didymin has been reported to have antioxidant and anti-cancer properties [39,40]. However, there is no document to show that it is benefit to metabolic diseases. So the effects of didymin on the metabolic disorders need to be dissected in future.

Previous studies have suggested that pomelo peel or pomelo peel extracts could prevent the development of metabolic syndromes. However, the underlying mechanisms involved in the preventive effects of pomelo extracts were poorly understood. Here, we use HF diet-fed C57BL/6 mice to study the effects of pomelo peel extracts on lipid and glucose metabolism and their possible underlying mechanisms. Because the liver and WAT play a very important role in glucose and lipid metabolism, the current study focused on the mRNA expression levels of related genes in these organs. The results showed that both LP and BB peel extracts increased the gene expression of PPARα and GLUT4 in liver and WAT. It has been demonstrated that PPARα activation or the increase of its expression may increase peripheral lipid clearance via elevated β-oxidation [41]. We showed that the extracts are rich in maringin and didymin. Naringin has been reported to activate PPARα [42]. However, there is no report to show that naringin increases the mRNA level of PPARα. So didymin may be responsible for the increase of PPARα mRNA, but it needs further investigation. The enhancement of PPARα signaling was verified by the expression of its downstream genes. The expression of PGC-1β, Cyp4a10, Cyp4a14 and CD36 were significantly increased in the liver and PGC-1α, ACC, and ACO were also increased in the WAT of mice supplemented with LP peel extract. Moreover, the expression of ACO and FAS were decreased in the liver of mice treated with LP and BB peel extracts. We noticed that LP increased the expressions of ACC, ACO, and CD36 in WAT, while BB did not. However, the LP only increased expression by about 10-30% which may be the reason for the discrepancy between LP and BB. These results suggest that pomelo peel extracts may improve hyperlipidemia via the elevation of PPARα expression in HF diet-fed mice.

GLUT4 is the insulin-regulated glucose transporter that plays an important role in stimulating glucose uptake into the cell. GLUT4 is primarily found in skeletal muscle, cardiac muscle and adipose tissues. It has been demonstrated that citrus flavonoid nobiletin improves hyperglycemia and insulin resistance by increased GLUT4 translocation to the plasma membrane in WAT and muscle [43]. Our results have shown that pomelo peel extracts increased the expression of GLUT4 in liver and WAT. Consequently, the beneficial effects of pomelo peel extracts on glucose intolerance and insulin resistance observed in the present study may contribute to increase the mRNA levels of GLUT4. However, a clearer understanding of the contribution of pomelo peel extracts to the control of metabolic disorders will require further molecular studies.

## Conclusion

In summary, we demonstrated that pomelo peel extracts had several beneficial effects on HF diet-fed mice, possibly though the activation of PPARα and GLUT4, resulting in prevention of the development of obesity induced by a HF diet, attenuation of dyslipidemia and hyperglycemia, improved glucose tolerance and insulin resistance. Our results indicate that pomelo peels could be used as a potential dietary therapy for metabolic disorders.
